# Thumbs up or thumbs down? Effects of neuroticism and depressive symptoms on psychophysiological responses to social evaluation in healthy students

**DOI:** 10.3758/s13415-016-0435-2

**Published:** 2016-05-10

**Authors:** F. M. van der Veen, M. J. W. van der Molen, M. W. van der Molen, I. H. A. Franken

**Affiliations:** 1 Institute of Psychology, Erasmus University Rotterdam, P.O. Box 1738, 3000DR Rotterdam, The Netherlands; 2 Institute of Psychology, Faculty of Social and Behavioral Sciences, Leiden University, Leiden, The Netherlands; 3 Leiden Institute for Brain and Cognition, Leiden University, Leiden, The Netherlands; 4 Department of Psychology, University of Amsterdam, Amsterdam, The Netherlands

**Keywords:** Social rejection, Social feedback, Neuroticism, Depressive symptoms, P3, Heart rate, Theta oscillations, Gender

## Abstract

The effects of neuroticism and depressive symptoms on psychophysiological responses in a social judgment task were examined in a sample of 101 healthy young adults. Participants performed a social judgment task in which they had to predict whether or not a virtual peer presented on a computer screen liked them. After the prediction, the actual judgment was shown, and behavioral, electrocortical, and cardiac responses to this judgment were measured. The feedback-related negativity (FRN) was largest after unexpected feedback. The largest P3 was found after the expected “like” judgments, and cardiac deceleration was largest following unexpected “do not like” judgments. Both the P3 and cardiac deceleration were affected by gender—that is, only males showed differential P3 responses to social judgments, and males showed stronger cardiac decelerations. Time–frequency analyses were performed to explore theta and delta oscillations. Theta oscillations were largest following unexpected outcomes and correlated with FRN amplitudes. Delta oscillations were largest following expected “like” judgments and correlated with P3 amplitudes. Self-reported trait neuroticism was significantly related to social evaluative predictions and cardiac reactivity to social feedback, but not to the electrocortical responses. That is, higher neuroticism scores were associated with a more negative prediction bias and with smaller cardiac responses to judgments for which a positive outcome was predicted. Depressive symptoms did not affect the behavioral and psychophysiological responses in this study. The results confirmed the differential sensitivities of various outcome measures to different psychological processes, but the found individual differences could only partly be ascribed to the collected subjective measures.

Social media have changed the way people interact in modern society. Many people use Facebook, Twitter, and Instagram to interact with friends, family, and more distant acquaintances. An important property of these tools is that they all have very direct options to instantly evaluate the input of others. In Facebook and Instagram we “like” the contributions of others, and in Twitter we retweet and comment. This constant social evaluation has a massive impact on how we interact, and more importantly on how we feel about ourselves and others (O’Keeffe & Clarke-Pearson, 2011). Social evaluation plays a major role in psychiatric illnesses such as major depression (Slavich, O’Donovan, Epel, & Kemeny, 2010), a link that deserves further study.

A paradigm to investigate the impact of social evaluation in the laboratory has been developed by Somerville and colleagues (Somerville, Heatherton, & Kelley, 2006). They created a task in which participants were asked to send a picture of themselves to the researchers and were told that a group of people would evaluate their picture on the basis of a first impression, in terms of “like” or “do not like.” In the actual task, participants saw the pictures of these virtual judges and were asked whether or not they thought this person liked them (version 1) or whether or not they liked the person shown in the picture (version 2). After giving their prediction (1) or evaluation (2), the evaluation (in both versions “like” or “do not like”) of the virtual judge was shown. Somerville et al. measured the brain response to this evaluation by using functional magnetic resonance imaging (fMRI) and found that the dorsal part of the anterior cingulate cortex (dACC) responded to the congruence between their own prediction/evaluation and the evaluation of the virtual judge, with more activation in the dACC for incongruent evaluations. Furthermore, they found that the ventral part of the ACC (vACC) was more active following positive evaluations. So, they concluded that one part of the ACC responds to whether or not an evaluation fits our prediction, and the other part of the ACC responds to the actual valence of the evaluation.

In more recent studies, the first version of this social judgment paradigm has been used to evaluate whether the two important properties (congruence and valence) of social evaluation can be measured in different output systems by using cardiovascular (Moor, Crone, & van der Molen, 2010) and event-related brain potential (ERP; Dekkers, van der Molen, Moor, van der Veen, & van der Molen, 2015; van der Veen, van der Molen, Sahibdin, & Franken, 2014) responses, and how the behavioral and psychophysiological responses develop during childhood (Moor, van Leijenhorst, Rombouts, Crone, & Van der Molen, 2010) and are modulated by anxiety (Van der Molen et al., 2013). In a first study, Moor, Crone, and van der Molen (2010) found that the cardiovascular system was sensitive to a combination of both the congruence and valence of the social judgment, as was shown by a stronger decelerative response to unexpected “do not like” judgments. This finding has been replicated a number of times (Dekkers et al., 2015; van der Veen et al., 2014) and has been related to activation of the dACC, which might possibly play the role of a neural alarm system activated by cues that signal social pain (Eisenberger & Lieberman, 2004). More recently, event-related brain responses have been examined in this task. In the first study, it was found that the P3 amplitude was largest for expected “like” judgments, and that the P3 amplitudes for the other categories did not differ, but were much smaller than for expected “like” judgments (van der Veen et al., 2014). The P3 is a positive-going ERP component that is maximal between 300 and 800 ms after stimulus onset, and in which P3a and P3b components are often distinguished. The P3a is thought to reflect “stimulus-driven frontal attention mechanisms during task processing,” and the P3b is thought to reflect “attention and appears related to subsequent memory processing” (Polich, 2007, p. 2128). The P3a peaks somewhat earlier and has a fronto-central distribution, as compared the later-peaking P3b, which has a more posterior distribution. The P3 in this task was interpreted as a P3a, and the finding of a larger P3 amplitude following expected “like” judgments was related to activation of the vACC and the motivational properties of positive social evaluations when one is expecting to get a positive social evaluation. In a later study, an enhanced P3 to expected acceptance was found in a very similar paradigm, although it was not explicitly reported (Sun & Yu, 2014). In the most recent study, however, the larger P3 for expected “like” judgments could not be replicated (Dekkers et al., 2015); however, it was found that although social evaluations resulted in larger P3 amplitudes than did nonsocial evaluations, this amplitude was not affected by the congruence or valence of the evaluation. In addition, in the latter study it was found that the congruence of judgment and expectation affected the feedback-related negativity (FRN), as measured at fronto-central locations. The FRN is a negative-going component peaking around 250 ms poststimulus, is largest when the outcome is incongruent with expectations, and has been related to performance monitoring (Ullsperger, Danielmeier, & Jocham, 2014). In the study by Dekkers et al., the FRN was largest for unexpected events and was interpreted to be related to the dACC activation described by Somerville et al. (2006) and seen as support for the *prediction of response–outcome* (PRO) theory (Alexander & Brown, 2010). According to the PRO theory, the main function of the ACC is the prediction of the most frequently occurring outcome, and the ACC sends out a signal when this outcome does not occur. In this way, the incongruence between expectation and outcome triggers the signal from the ACC (i.e., a larger FRN), irrespective of valence. Similar findings were recently reported in a study that examined the influence of fear of negative evaluation (Van der Molen et al., 2013), in which a strong effect of congruence on FRN amplitudes was found, but no effect of congruence or valence on P3 amplitudes.

The discrepancy between the ERP findings reported in various studies is not easy to explain. Possibly the use of a control task and gender differences might explain the lack of P3 task effects in some of these studies (Dekkers et al., 2015; Van der Molen et al., 2013). In the study of van der Veen et al. (2014), both males and females were included, whereas in the studies of Van der Molen et al. and Dekkers et al., only females were included. Earlier research (Benenson et al., 2013) had shown that males and females differ in both their uses of and responses to social exclusion, a concept strongly related to the negative social feedback given in the social judgment task. On the other hand, relatively small samples have sometimes been used. In the present study, these issues were tackled by performing an experiment that included large numbers of both females and males. The second discrepancy concerns the reported FRN findings, which were absent in the van der Veen et al. study. As can be seen in the figures of both the Van der Molen et al. and Dekkers et al. studies, the FRN is hard to quantify, due to its overlap with the following P3. The FRN can be quantified in a number of different ways, and different methods are known to lead to different outcomes (Banis & Lorist, 2012). Furthermore, ERPs are less sensitive to the electrocortical dynamics that govern FRN activity, and time–frequency analysis might provide more insight (Cohen, 2011; Makeig, Debener, Onton, & Delorme, 2004). In this study, we aimed at quantifying the process underlying the FRN by means of the power of theta oscillations. Theta oscillations are thought to be responsive to the same underlying processes as the FRN, but are thought to reflect the activation of the underlying brain systems more closely (Cohen, Elger, & Ranganath, 2007). A recent study has associated the amplitude of theta oscillations with social exclusion (Cristofori et al., 2013), a process closely related to social rejection as measured in the social judgment paradigm. Finally, large individual differences in the behavioral and psychophysiological responses have been found in this paradigm (van der Veen et al., 2014), which might be related to personality traits and psychopathology. Due to the suggested relation with depression (Ayduk, Downey, & Kim, 2001; Slavich et al., 2010), we aimed at exploring, by means of questionnaires, the relations of the responses with neuroticism, a possible vulnerability factor for developing major depression (Roberts & Kendler, 1999), and depressive symptoms.

In the present study, we examined the influences of subjectively reported depressive symptoms and neuroticism on behavioral and psychophysiological responses in the social judgment task. In line with our previous study (van der Veen et al., 2014), we expected that the largest P3 would be found at fronto-central locations and for expected “like” judgments, and the largest cardiac decelerations would be found for unexpected “do not like” judgments. Furthermore, it was expected that unexpected judgments would lead to the largest theta oscillations, on the basis of the assumption that theta oscillations closely resemble the FRN response in feedback tasks (Cohen et al., 2007) and the earlier-reported congruence effects reported in the social judgment task (Dekkers et al., 2015; Van der Molen et al., 2013). Due to the suggested relation between reward and delta oscillations (Cohen, Elger, & Fell, 2009) and the relation between delta oscillations and P3 amplitudes (Basar, Basareroglu, Rosen, & Schutt, 1984), we expected the highest delta power following expected “like” judgments, which were found to be associated with the largest P3 amplitude and can be seen as the most rewarding stimuli (van der Veen et al., 2014). On the basis of findings in feedback and reward studies, we expected that higher neuroticism scores would be related to higher theta power for especially unexpected judgments (Mueller et al., 2014), and a larger cardiac response to unexpected rejection (Mueller, Stemmler, Hennig, & Wacker, 2013). For depressive symptoms and neuroticism, we expected to find that higher depressive symptoms and neuroticism scores would be related to a more negative prediction bias (Beck, 1979), as reflected in a higher percentage of “do not like” predictions. On the basis of findings with respect to the FRN (Cavanagh, Bismark, Frank, & Allen, 2011; Mies et al., 2011; Santesso et al., 2008; Tucker, Luu, Frishkoff, Quiring, & Poulsen, 2003), we expected that higher depressive symptom scores would be associated with stronger theta power.

## Method

### Participants

A total of 131 participants were tested, of which 104 participants (mean age 20.9, *SD* = 2.3; 56 females, 48 males) had complete data sets, including all subjective, psychophysiological, and behavioral measures. Participants signed a written informed consent and were paid a small amount of money (€10) or received course credit. Participants were screened with a general health questionnaire and were excluded when major health problems that would interfere with task performance or the outcome measures were reported. Exclusion criteria were the presence of any neurological or psychiatric illness. This study was performed according to the local ethical guidelines of the Institute of Psychology at Erasmus University Rotterdam.

### Stimuli and procedure

Participants performed the social judgment task, which was based on the paradigm developed by Somerville et al. (2006). The participants were instructed by using a cover story. At least one week before the actual experiment, they were asked to send a picture of themselves to the experimenters. At the same time, they completed a number of questionnaires (see the subjective measures) using Qualtrics (Qualtrics, Provo, UT). The participants were told that the picture they had sent would be judged by a panel of peers participating in a larger social experiment at a different university in the Netherlands. The judgment would be based on a first impression and would be formulated in terms of “like” or “do not like.” Participants were told that this judgment would be sent back to the experimenters alongside the picture of the panel member. In the actual experiment participants performed two tasks, of which only the social judgment task is described in this article. After general instructions and signing informed consent, they were attached to the electroencephalographic (EEG) equipment. In the social judgment task, participants were asked to look at the pictures of these panel members, which were presented on a computer screen. They were instructed to predict whether or not the person shown on the screen would like them. After giving their prediction, the actual judgment was presented. Stimuli were presented using the E-Prime software (Psychology Software Tools, Pittsburgh, PA). In the task, the participants viewed 120 faces with a neutral face expression, derived from the Nimstim (Tottenham et al., 2009), KDEF (Lundqvist, Flykt, & Öhman, 1998), and RAFD (Langner et al., 2010) emotional face databases. Pictures from different databases were transformed to similar sizes; the selection of pictures consisted of 50 % male and 50 % female faces, and the faces were presented in black and white against a black background. Participants had to decide whether or not they thought the presented person liked them by pressing with their right hand either the leftmost (Yes) or the rightmost (No) button on a standard five-key E-Prime response panel. Trials started with the onset of the face, which was presented for a fixed period of 6 s. After the onset of the face, participants were required to provide their answer within a 3-s response window. After these 3 s, the given answer (yes/no) of the participant was presented on the left side of the face of the panel member. After another second, the actual evaluation of the panel member was presented on the right side of the screen (the participant’s prediction was still visible). The participants were not actually evaluated by the persons presented in the task, but the evaluations were based on a computer-generated quasirandom sequence consisting of 50 % “like” (yes/“Ja”) and 50 % “do not like” (No/“Nee”) evaluations. The same sequence of evaluations was presented to every participant, so the only thing that differed between participants was the prediction of the participant. After finishing the task, participants were debriefed about the evaluations and the goal of the experiment.

### Questionnaires

Neuroticism was assessed with the revised and shortened 48-item Dutch version of the Eysenck Personality Inventory (EPQ-rss; Eysenck & Eysenck, 1975; Sanderman, Eysenck, & Arrindell, 1991). The EPQ-rss is a self-report questionnaire in which 12 yes/no questions measure neuroticism. The internal consistency of the Neuroticism scale is good, with a Cronbach’s *α* of .80 in the present sample. Depressive symptoms were measured with the Dutch version of the Beck Depression Inventory (BDI; Beck, Steer, & Garbin, 1988; Bosscher, Koning, & Van Meurs, 1986). The BDI is a self-report questionnaire that is used to measure the level of depression. It consists of 21 items that measure different psychological and somatic symptoms of depression. Participants rate themselves on a 4-point Likert scale (0–3), with 0 indicating the *absence* of a symptom, and 3 referring to *intense presence* of the symptom. The internal consistency of this list is also good, with a Cronbach’s *α* of .84 in the present sample.

### EEG signal recording

The EEG was recorded with BioSemi Active-Two using 33 channels (10–20 System, and one additional electrode at FCz) with Ag/AgCl active electrodes mounted in an elastic cap. An electrocardiogram (ECG) was recorded from a single lead placed below the left ribcage. Signals were recorded with a low-pass filter of 134 Hz and were digitized with a sample rate of 512 Hz and 24-bit analog/digital conversion. The signals were referenced offline to mathematically linked mastoids. A vertical electrooculogram (EOG) was derived from electrodes placed above and below the left eye. A horizontal EOG was derived from electrodes next to each eye. BioSemi uses the common mode sense (CMS) and driven right-leg electrodes to create a feedback loop that replaces the conventional ground electrode. The CMS was used as an online reference. The EEG and ECG data were analyzed offline using Vision Analyzer (Brain Products GmbH, Munich, Germany).

### EEG preprocessing

For both the power and ERP analyses, the EEG signals were filtered using a band-pass filter between 1 and 40 Hz (phase-shift-free Butterworth filters, 24 dB/octave slope). The EEG signal was locked to the onset of the feedback stimulus, and epochs were extracted between 2,000 ms preceding and 2,000 ms following the onset of this stimulus. The epochs were corrected for EOG artifacts by using ocular independent component analysis (ICA) as implemented in Brain Vision Analyzer. To optimize the data, ICA segments containing large artifacts were removed using a visual inspection method. Next, automatic artifact removal was applied by removing segments containing large voltage steps (>50 *μ*V), a large difference between maximum and minimum (>200 *μ*V), absolute values exceeding 1 mV, and activity below 0.5 *μ*V. After correction, averages of 29.8 ± 0.81 (mean ± *SEM*) trials were kept for expected positive feedback, 29.8 ± 0.77 for unexpected negative feedback, 25.9 ± 0.76 for unexpected positive feedback, and 24.0 ± 0.74 for expected negative feedback.

### Event-related brain potentials

For the ERP analysis, a 200-ms prefeedback period was used for baseline correction. Visual inspection of the grand average ERPs showed that the P3 amplitude was maximal between 300 and 400 ms after stimulus onset, and therefore we decided to quantify the P3 amplitude as the average voltage in the area between 300 and 400 ms after the stimulus onset. The FRN peak amplitude was computed by first identifying the P2 amplitude—the most positive value in the 150- to 250-ms postfeedback window—as the onset of the negativity. Second, we determined the most negative value within a window from 200 until 350 ms postfeedback, and finally, took the difference between the P2 amplitude and this most negative value as the FRN.

### Time–frequency analysis

For the time–frequency analysis, the data were transformed with a current source density transformation, after which the theta oscillations at electrode FCz were analyzed using a continuous wavelet transformation, as implemented in BrainVision Analyzer (Morlet complex waveform, frequency range from 1 to 40 Hz in 40 logarithmic steps, Morlet parameter *c* = 7).

The time–frequency neuronal oscillatory power was extracted from the data by convolution of the single trials with complex Morlet wavelets, which can be defined as Gaussian-windowed sine waves:

1 $$ \varPsi \left(t,f\right)=A{e}^{-{t}^2/\left(2{\sigma}_t^2\right)}\times {e}^{i2\pi ft}, $$

where Ψ denotes the complex convolution with the wavelet function, *t* is time, and *f* is the frequency, which increased from 1 to 40 Hz in 40 logarithmically spaced steps. *A* represents the normalization function, which normalizes the wavelet function so that all frequencies have the same energy value of 1 and allows for comparisons of the signal across all frequency levels, and *σ*_*t*_ represents the standard deviation of the Gaussian bell function. The Morlet parameter *C* = *f*(2*πσ*_*t*_) was set to 7. Subsequently, estimates of power were extracted from the complex signal resulting after convolution of the complex Morlet wavelet with the single-trial data: *p*(*t*) = (real[*z*(*t*)]^2^ + imag[*z*(*t*)]^2^). The cue-locked power was thereafter normalized with a percent change from baseline (i.e., –400 to –100 ms prior to the onset of the feedback stimulus), since power decreases with increasing frequencies (power law). The total power was calculated by averaging across trials and exported separately for the delta (2–3 Hz) and theta (5–7 Hz) oscillations.

### Interbeat intervals

R peaks were detected in the ECG signal using the peak detection algorithm implemented in BrainVision Analyzer, and interbeat intervals (IBIs) were computed between consecutive *r* peaks. Missing values and artifacts were detected by visual inspection and corrected manually. We selected six IBIs surrounding the judgment stimulus for further analysis—that is, two preceding IBIs (–2 and –1), the current IBI (i.e., IBI 0), and the three subsequent IBIs (i.e., IBIs 1, 2, and 3). As in our previous study, IBIs 0 to 3 were referenced to the second IBI preceding stimulus onset (IBI –2).

### Statistical analysis

Behavioral and electrocortical measures were statistically evaluated using SPSS 18 (SPSS Inc., Chicago, IL). Analysis of variance was performed using a general linear model (GLM) repeated measures design. For the behavioral measures, we analyzed the prediction bias quantified as the percentage of expectations of getting a “like” judgment. Bias was tested using a one-sample *t* test with 50 % as a criterion. Bivariate Pearson correlations were computed between bias, neuroticism, and depressive symptoms scores. The P3 and FRN amplitudes were tested in a design with Electrode Position (three levels: Fz, Cz, and Pz), Expectation (two levels: “Like” vs. “Do Not Like”), and Feedback (two levels: “Like” vs. “Do Not Like”) as within-subjects factors. Visual inspection of the wavelet data showed that both delta and theta power were maximal on the FCz electrode. Theta was maximal between 200 and 500 ms poststimulus, and delta was maximal between 100 and 700 ms poststimulus. Theta power was quantified by computing the average power in the theta band (5–7 Hz) between 200 and 500 ms after stimulus onset at FCz. Delta power was quantified by computing the average power in the theta band (2–3 Hz) between 100 and 700 ms after stimulus onset at FCz. Theta power was tested in a design with Expectation and Judgment as within-subjects factors. Interbeat intervals were tested in a design with Expectation, Judgment, and Sequential IBI (four levels: 0, 1, 2, and 3) as within-subjects factors. After performing a first repeated measures GLM analysis without covariates, we performed additional GLM analyses for all physiological variables in which neuroticism and depressive symptoms were entered sequentially as covariates. These scores were centered using the method of Delaney and Maxwell (1981), because of a large sum-of-squared error resulting from the addition of these measures as a covariate in the analyses. We used the mean minus the mean of all participants (Delaney & Maxwell, 1981; Thomas et al., 2009). Huynh–Feldt corrections of the degrees of freedom were applied whenever appropriate, but uncorrected degrees of freedom are reported. Effects size is reported as partial eta squared (*η*_p_^2^). Follow-up analyses were performed whenever significant interactions were found, and the *p* values of these tests were Bonferroni-corrected.

## Results

### Subjective measures

The average score on the BDI was 5.3 (minimum = 0, maximum = 28; *SEM* = 0.56), and 12 participants (12 %) scored 14 or higher, which is considered to be the cutoff score for mild depression. The average score for neuroticism was 3.8 (minimum = 0, maximum = 12; *SEM* = 0.303). The neuroticism and BDI scores were strongly positively correlated, *r* = .490, *p* < .001.

### Performance

On the basis of a box-plot analysis, one participant was labeled an outlier and was excluded from further analysis. Due to too many artifacts in the EEG analysis, two additional participants had to be excluded from the analyses, leaving a total of 101 participants who could be analyzed for all measures. The scores for prediction bias differed significantly from 50 %, *t*(100) = 4.5, *p* < .0005. Generally speaking, the participants showed a positive bias, and expected to be liked on 55 % ± 1.1 % (mean ± *SEM*) of the trials. There was no significant correlation with the BDI score, *r* = .003, *p* = .978, but a significant correlation with neuroticism was observed, *r* = –.211, *p* = .034. This correlation shows that higher neuroticism scores were associated with a more negative bias score. The biases did not differ between males (55 %) and females (55 %), *p* = .940.

### FRN

**Fig. 1:**
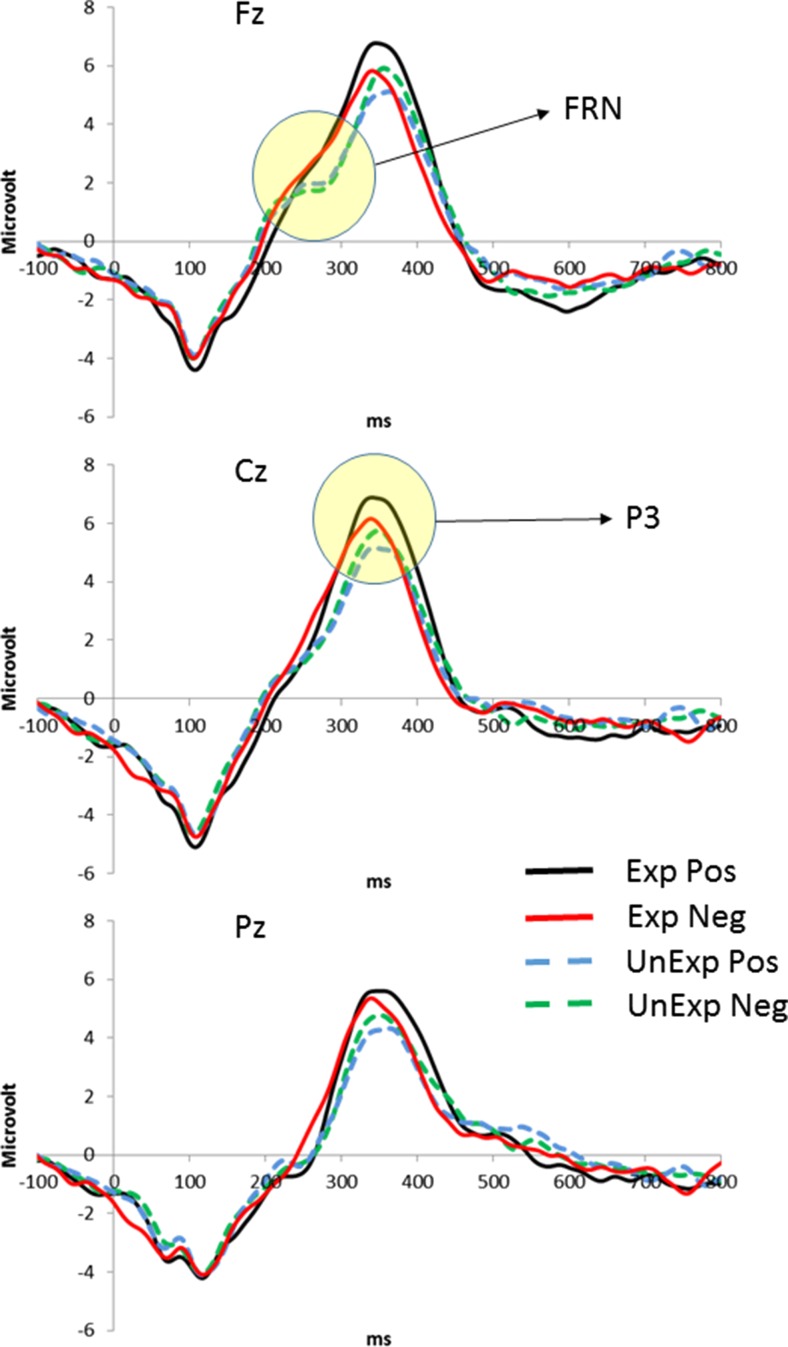
Grand average waveforms for Fz, Cz, and Pz and for expected (Exp) and unexpected (UnExp) positive (Pos) and negative (Neg) social feedback. Circles identify the FRN and P3 components on the waveforms

**Fig. 2:**
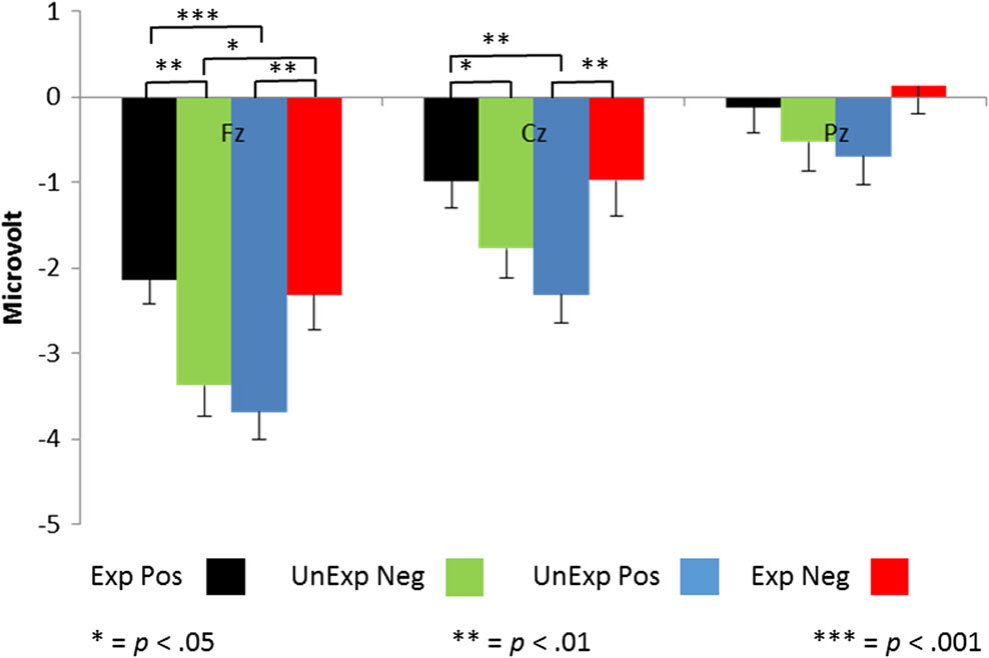
FRN amplitudes, as measured at Fz, Cz, and Pz for expected (Exp) and unexpected (UnExp) positive (Pos) and negative (Neg) social feedback. See the text for further details

The grand average waveforms of the ERPs at the three tested electrode positions are shown in Fig. 1. Statistical analysis of the FRN amplitudes showed a main effect of electrode position, *F*(2, 200) = 110.5, *p* < .0005, *η*_p_^2^ = .525. Furthermore, an interaction between expectation and feedback, *F*(2, 200) = 12.4, *p* = .001, *η*_p_^2^ = .110, was found. Finally, we observed a three-way interaction between all of the factors, *F*(2, 200) = 6.9, *p* = .005, *η*_p_^2^ = .064. Follow-up analyses showed that the FRN amplitude was larger at Fz (*M* = –2.9 *μ*V) than at Cz (*M* = –1.5 *μ*V), *p* < .0005, and Pz (*M* = –0.3 *μ*V), *p* < .0005, and larger at Cz than at Pz, *p* < .0005. Furthermore, we found that the FRN was larger for both unexpected positive (*M* = –2.2 *μ*V) and unexpected negative (*M* = –1.9 *μ*V) judgments, as compared to both expected positive (*M* = –1.1 *μ*V), *p* = .001, and negative (*M* = –1.1 *μ*V), *p* = .037, judgments. Further follow-up analyses showed that the FRN was larger at Fz for both unexpected positive (*M* = –3.7 *μ*V) and unexpected negative (*M* = –3.4 *μ*V) judgments, as compared to expected positive (*M* = –2.1 *μ*V), *p* < .0005, and negative (*M* = –2.3 *μ*V), *p* = .018, judgments. At Cz, the FRN was only larger for unexpected positive judgments (*M* = –2.3 *μ*V) than for expected positive judgments (*M* = –1.0 *μ*V), *p* = .001, and at Pz no significant differences were found. For a summary of these FRN results, see Fig. 2. In the analyses in which the neuroticism and depressive symptom scores were entered sequentially as covariates, no main or interaction effects were found of these covariates. In a final analysis, we entered Gender as a between-subjects factor. In this analysis, a main effect of gender, *F*(1, 99) = 6.2, *p* < .05, *η*_p_^2^ = .059, and a three-way interaction between gender, electrode position, and feedback, *F*(2, 198) = 12.9, *p* < .0005, *η*_p_^2^ = .115, were found. Females (*M* = –2.0 *μ*V) showed a larger FRN than males (*M* = –1.0 *μ*V). Further follow-up analyses showed that this effect was only significant for both negative (*M* = –3.4 *μ*V vs. *M* = –2.3 *μ*V), *p* = .007, and positive (*M* = –3.6 *μ*V vs. *M* = –1.9 *μ*V), *p* = .030, judgments at Fz, and for positive judgments at Cz (*M* = –2.3 *μ*V vs. *M* = –1.0 *μ*V), *p* = .014, and Pz (*M* = –1.0 *μ*V vs. *M* = 0.2 *μ*V), *p* = .022. To summarize, FRN amplitudes were not affected by neuroticism and depressive symptoms scores, but were affected by gender.

### P3

**Fig. 3:**
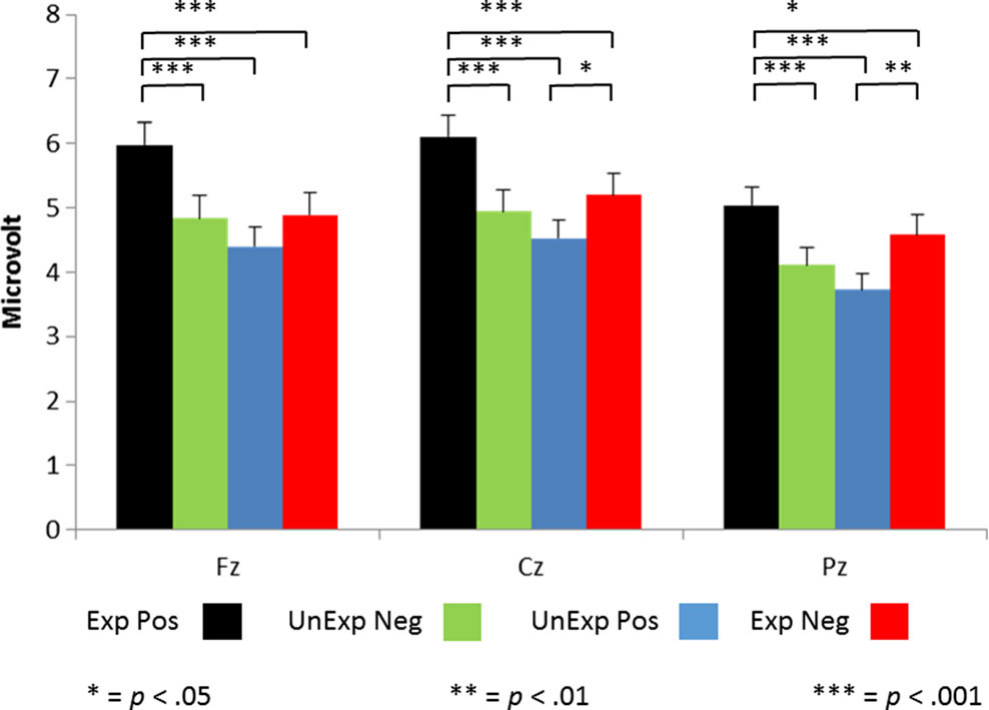
P3 amplitudes, as measured at Fz, Cz, and Pz for expected (Exp) and unexpected (UnExp) positive (Pos) and negative (Neg) social feedback. The P3 amplitude is quantified as the average amplitude in an area between 300 and 400 ms poststimulus

P3 amplitudes were first analyzed without covariates, in a model with Expectation, Feedback, and Electrode Position as within-subjects factors. We found main effects of electrode position, *F*(2, 200) = 23.9, *p* < .0005, *η*_p_^2^ = .193, and expectation, *F*(1, 100) = 13.1, *p* < .0005, *η*_p_^2^ = .116, as well as two-way interactions between expectation and feedback, *F*(1, 100) = 20.5, *p* < .0005, *η*_p_^2^ = .170; between electrode position and expectation, *F*(2, 200) = 5.6, *p* = .012, *η*_p_^2^ = .053; and between electrode position and feedback, *F*(2, 200) = 4.6, *p* = .023, *η*_p_^2^ = .044. Follow-up analyses showed that the P3 amplitude was higher at Cz (*M* = 5.2 *μ*V) than at Pz (*M* = 4.3 *μ*V), *p* < .0005, and was also significantly higher at Fz (*M* = 5.0 *μ*V) than at Pz, *p* < .0005. P3 amplitudes were also higher for expected positive judgments (*M* = 5.2 *μ*V) than for expected negative judgments (*M* = 4.6 *μ*V). Further follow-up analyses showed that for positive judgments, the P3 amplitude was higher when the judgments were expected (*M* = 5.7 *μ*V) than when they were unexpected (*M* = 4.6 *μ*V), *p* < .0005. For negative judgments, expectation did not influence the P3 amplitude. P3 amplitudes were higher when a positive judgment was expected at Fz (*M* = 5.5 *μ*V vs. *M* = 4.6 μV), *p* < .0005; Cz (*M* = 5.5 *μ*V vs. *M* = 4.9 *μ*V), *p* = .001; and Pz (*M* = 4.6 *μ*V vs. *M* = 4.2 *μ*V), *p* = .012, but the effect was strongest at Cz and Fz. The P3 amplitude was higher when a positive judgment was given, but this effect was not significant at any of the electrodes. The P3 amplitudes for the different stimulus categories and electrode positions are shown in Fig. 3. In the analyses in which neuroticism and depressive symptoms scores were entered sequentially as covariates, no main or interaction effects were found of these covariates. In a final analysis, we entered Gender as a between-subjects factor. In this analysis, a two-way interaction between gender and judgment, *F*(1, 100) = 5.7, *p* = .019, *η*_p_^2^ = .054, was found. Follow-up analyses showed that only in males did feedback affect the P3 amplitude. Male participants showed a reduced P3 amplitude for negative judgments (*M* = 4.9 *μ*V), as compared to positive judgment (*M* = 5.5 *μ*V), *p* = .010, whereas female participants did not show this difference (*M* = 4.7 *μ*V vs. *M* = 4.5 *μ*V). To summarize, depressive symptoms and neuroticism did not affect P3 amplitudes in the present study, but gender did.

### Theta power

**Fig. 4:**
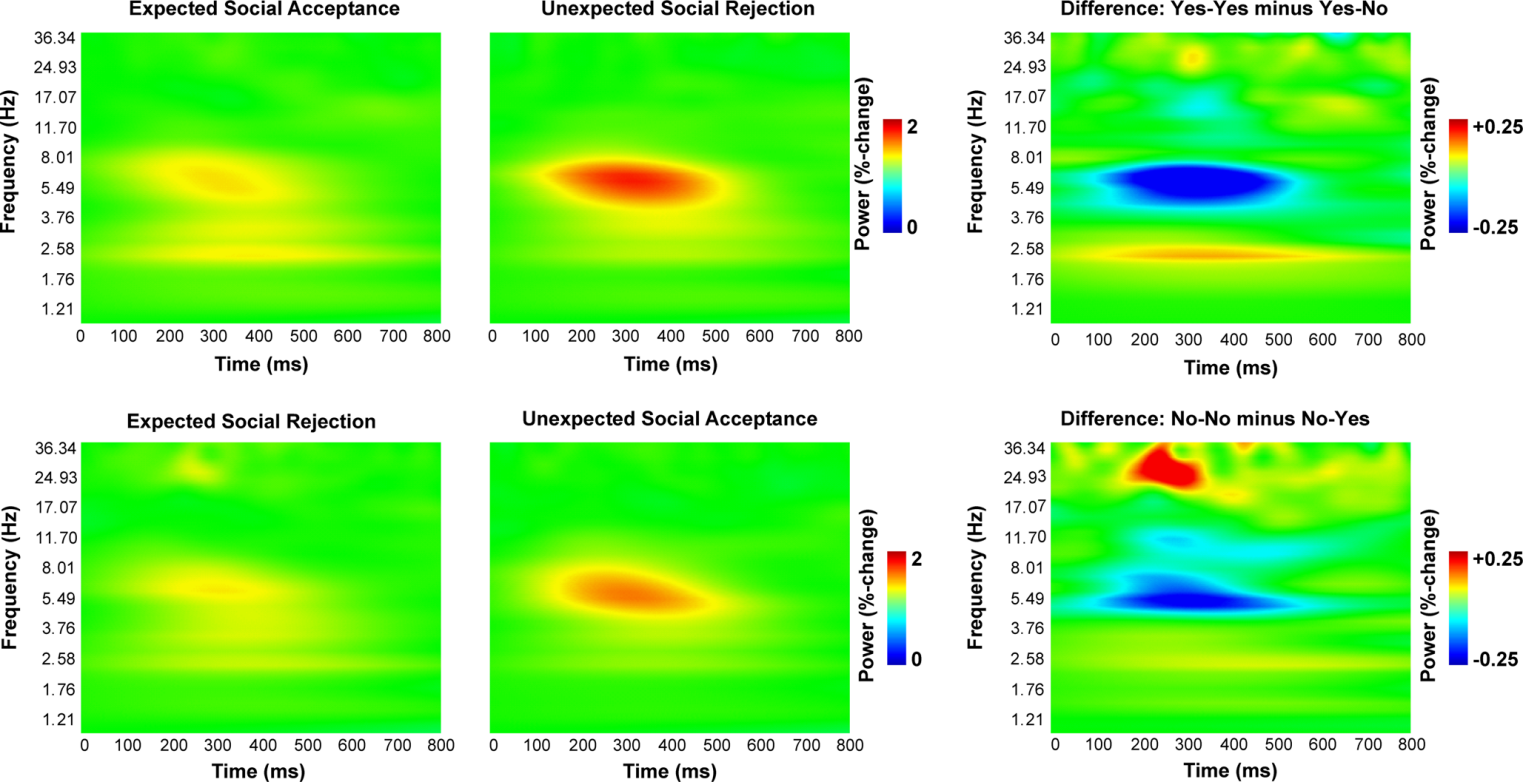
Total time–frequency power, as measured at FCz for expected and unexpected positive and negative social feedback. The rightmost two graphs represent the difference between expected positive (Yes–Yes) and unexpected negative (Yes–No) feedback and the difference between expected negative (No–No) and unexpected positive (No–Yes) feedback

**Fig. 5:**
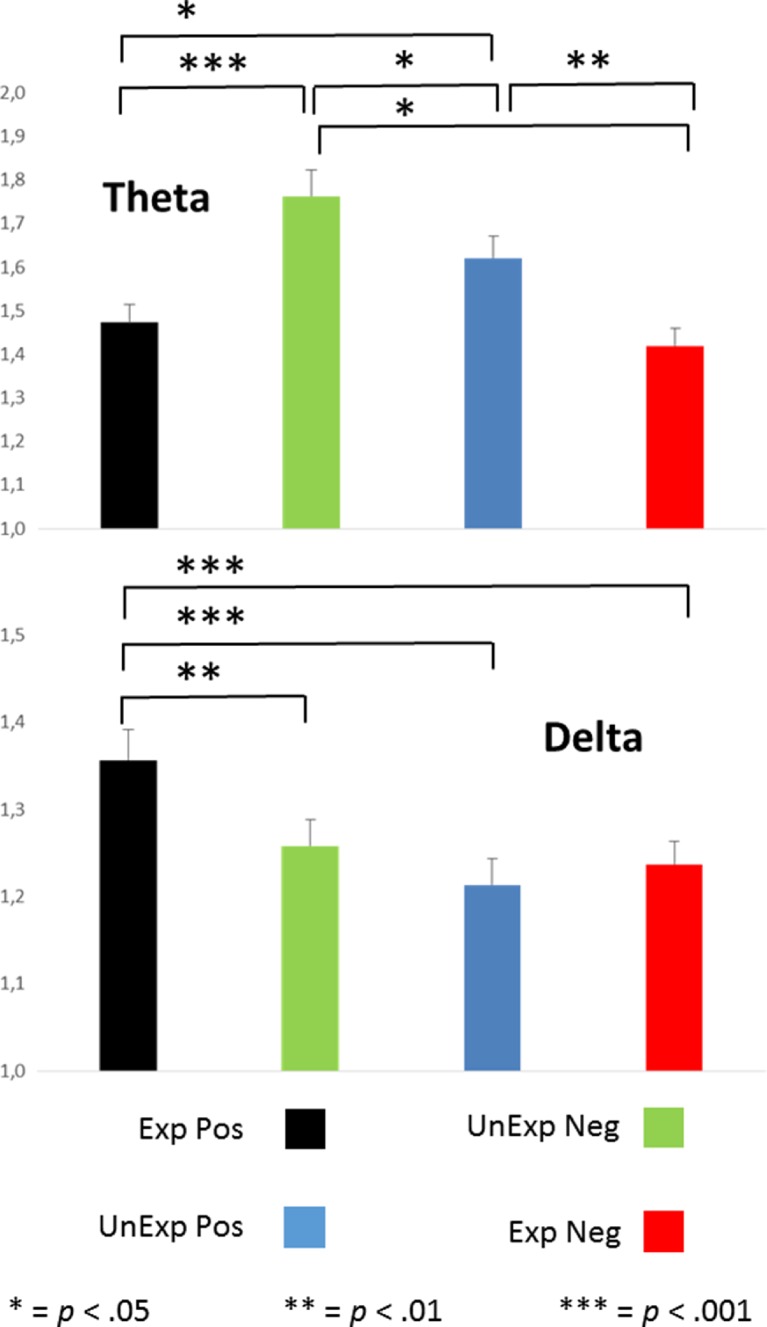
Total theta and delta power, as measured at FCz for expected (Exp) and unexpected (UnExp) positive (Pos) and negative (Neg) social feedback. See the text for details

Time–frequency data are shown in Fig. 4, and the specific results for both theta and delta power can be found in Fig. 5. Statistical analyses showed a main effect of expectation, *F*(1, 100) = 5.8, *p* = .018, *η*_p_^2^ = .055, and a two-way interaction between expectation and feedback, *F*(1, 100) = 28.6, *p* < .0005, *η*_p_^2^ = .223. Judgments preceded by an expectation not to be liked were associated with slightly more theta power (*M* = 1.62 vs. *M* = 1.52). Follow-up analyses showed that for “like” expectations, theta power was higher for “do not like” judgments (*M* = 1.47 vs. *M* = 1.76, *p* < .0005), and for “do not like” expectations, theta power was higher for “like” judgments (*M* = 1.62 vs. *M* = 1.42, *p* = .001). In other words, the congruence between expectations and judgments seems to be the main factor influencing theta power. Analyses with depressive symptoms and neuroticism as covariates did not yield additional effects, nor did adding Gender as a between-subjects factor. In other words, theta power was not influenced by gender, depressive symptoms, or neuroticism.

### Delta power

The statistical analyses of delta power showed a main effect of expectation, *F*(1, 100) = 11.5, *p* = .001, *η*_p_^2^ = .103, and a two-way interaction between expectation and feedback, *F*(1, 100) = 6.7, *p* = .011, *η*_p_^2^ = .063. Judgments preceded by the expectation to be liked were associated with slightly more delta power (*M* = 1.31 vs. *M* = 1.23). Follow-up analyses showed that for “like” expectations, delta power was higher for “like” judgments (*M* = 1.36 vs. *M* = 1.26, *p* = .007), whereas for “do not like” expectations, delta power did not differ between judgments (*M* = 1.21 vs. *M* = 1.24, *p* > .5). Analyses with neuroticism and depressive symptoms as covariates did not yield additional effects. The final analysis with Gender as a between-subjects factor showed a main effect of gender, *F*(1, 100) = 7.3, *p* = .008, *η*_p_^2^ = .069, caused by a higher delta power in males than in females (*M* = 1.33 vs. *M* = 1.21).

### Interbeat intervals

**Fig. 6:**
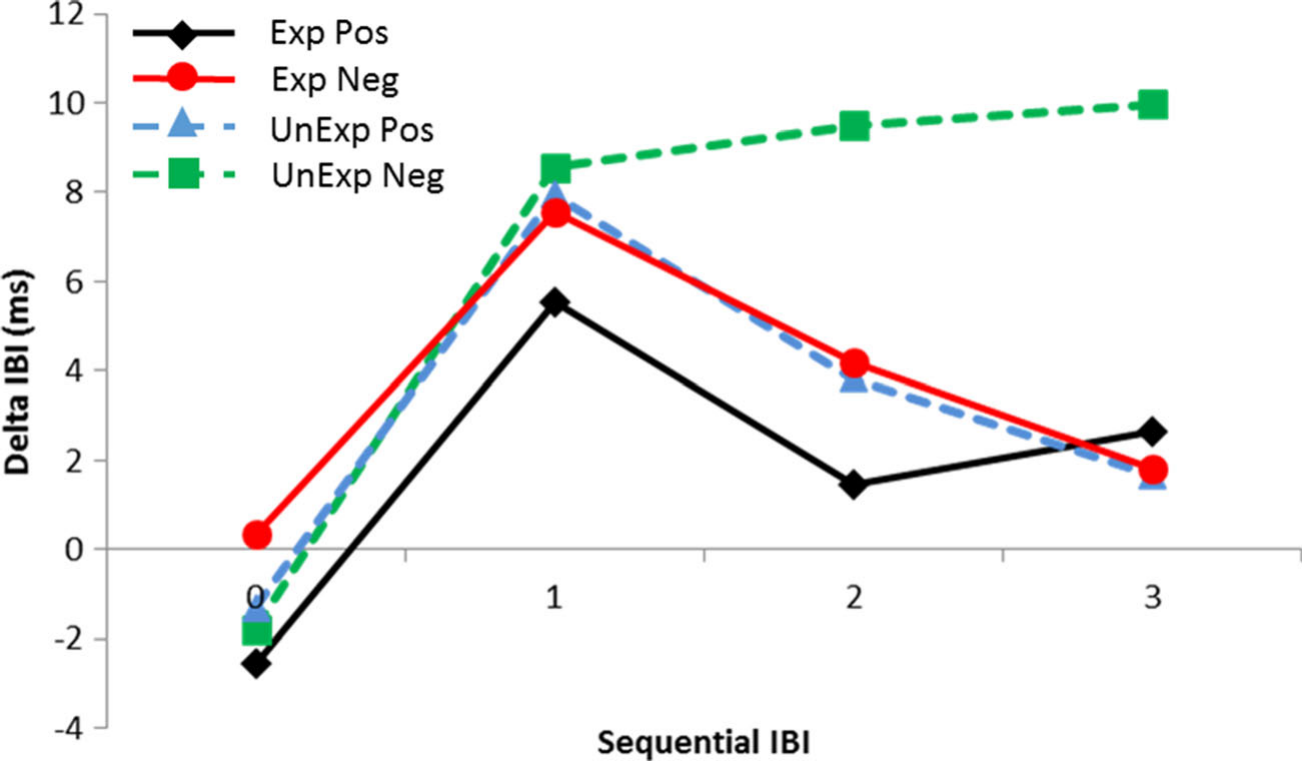
Cardiac responses for expected (Exp) and unexpected (UnExp) positive (Pos) and negative (Neg) social feedback. Cardiac responses are shown for IBIs 0, 1, 2, and 3 and are referenced to IBI –2

Cardiac responses are shown in Fig. 6. Analysis of the cardiac response without the covariates showed a main effect of sequential IBI, *F*(3, 300) = 18.5, *p* < .0005, *η*_p_^2^ = .156; two-way interactions between expectation and sequential IBI, *F*(3, 306) = 8.5, *p* < .0005, *η*_p_^2^ = .079, and feedback and sequential IBI, *F*(3, 306) = 4.7, *p* < .01, *η*_p_^2^ = .045; and a three-way interaction between all factors, *F*(3, 306) = 6.5, *p* < .005, *η*_p_^2^ = .061. Follow-up analyses showed that IBI 0 differed significantly from all other IBIs (*p* < .0005, *p* < .0005, and *p* = .003, respectively, as compared with IBIs 1–3), but no other comparisons yielded significant effects. Further follow-up analyses showed that only for judgments in which the participants expected a “like” judgment, and only for IBI 2, *p* < .0005, and IBI 3, *p* = .001, a larger cardiac deceleration was found for a “do not like” than for a “like” judgment. In the analyses in which neuroticism and depressive symptoms scores were entered sequentially as covariates, no main or interaction effects were found of the BDI score. For neuroticism, we found a three-way interaction between expectation, sequential IBI, and neuroticism scores, *F*(3, 300) = 3.4, *p* = .027, *η*_p_^2^ = .033. Follow-up correlation analyses showed that neuroticism scores only correlated significantly, *r* = –.212, *p* = .033, with expectations to be liked on IBI 3. Contrary to our expectations, smaller decelerations were associated with higher neuroticism scores. In a final analysis, we entered Gender as a between-subjects factor. In this analysis we found significant three-way interactions between gender, sequential IBI, and expectation, *F*(3, 300) = 3.6, *p* = .021, *η*_p_^2^ = .035, and between gender, expectation, and feedback, *F*(3, 303) = 5.0, *p* = .027, *η*_p_^2^ = .048. Follow-up analyses showed that, only for males and only when they received a “like” judgment, this judgment led to a smaller cardiac deceleration when it was expected (*M* = –1.8 ms) than when it was unexpected (*M* = 3.8 ms), *p* < .026. Further follow-up analyses showed that, only for males and only for IBIs 1 and 3, cardiac deceleration differed significantly between the different expectations. Deceleration was larger when a “do not like” judgment was expected at IBI 1 (*M* = 2.2 ms vs. *M* = 6.1 ms), *p* = .045, and also larger when a “like” judgment was expected at IBI 3 (*M* = 5.4 ms vs. *M* = –0.3 ms), *p* = .017. To summarize, depressive symptoms did not affect the cardiac response, but both neuroticism and gender significantly modulated that response.

### Correlations

We computed correlations between our behavioral and psychophysiological measures. Our findings showed that the expected “like” judgments and unexpected “do not like” judgments were the most informative, and therefore we decided to compute the difference between these two conditions for our various measures. For the P3, the difference was computed at Cz. For the FRN and the theta and delta oscillations, the difference was computed at FCz, and for cardiac deceleration, the difference was computed at IBI 2. None of the correlations were significant, except for the correlation between the P3 amplitude and delta oscillations, *r* = .328, *p* = .001, in which larger condition differences between P3 amplitudes were associated with larger condition differences between delta oscillations.

## Discussion

The main question in this study was whether behavioral and psychophysiological responses to social evaluative feedback were associated with neuroticism and depressive symptoms. The secondary aim of the study was to examine the usefulness of neural oscillatory dynamic measures in this paradigm. We found that depressive symptoms as measured with the BDI were not associated with the psychophysiological responses to negative social feedback. Neuroticism, on the other hand, was associated with both the expectancy bias and the cardiac response to social feedback. Participants scoring higher on neuroticism showed a more negative expectancy bias and, unexpectedly, a smaller cardiac decelerative response to social judgment stimuli in which a “like” judgment was expected. With respect to oscillatory responses, we found that theta power was related to the expectedness of the response and closely resembled the FRN. Delta power, on the other hand, was highest for expected “like” judgments and closely resembled the P3 amplitude. Neither delta nor theta power was related to the subjective measures.

The prediction bias scores and condition effects on both P3 amplitudes and cardiac responses were in line with those from the studies that have used this task previously. Like most previous studies (Moor, Crone, & van der Molen, 2010; Moor, van Leijenhorst, et al., 2010; Van der Molen et al., 2013; van der Veen et al., 2014), we found a slightly positive bias, which shows that people have a tendency to think that unknown people will judge them in a positive way, which is in line with the *person positivity effect* or *Polyanna effect* (Matlin & Stang, 1978).

As expected, the FRN amplitude was largest for incongruent social feedback stimuli. This finding is in line with two earlier studies using the same paradigm (Dekkers et al., 2015; Van der Molen et al., 2013). In these studies, it was suggested that the FRN response can be seen as a reflection of a neural mechanism involved in the early detection of incongruence. This interpretation is in line with the PRO model (Alexander & Brown, 2010), in which incongruence between expectation and outcome is seen as the major source of variance of the FRN, irrespective of valence.

The P3 amplitude was largest for expected “like” judgments on Cz, which is in accordance with our previous study using this task (van der Veen et al., 2014). Our findings with respect to P3 amplitudes, however, seem to be at odds with two other studies that have examined ERP responses in this task (Dekkers et al., 2015; Van der Molen et al., 2013), which reported no condition effects on P3 amplitudes. It should be noted, however, that these two studies did find that the P3 was largest for expected positive social feedback stimuli, but the effects were not significant. The lack of significant differences in these studies was possibly related to differences with respect to the tested samples. For the present study, we used a sample of both male and female participants, whereas the studies that did not find condition effects had tested only females. Social exclusion, which is closely related to the negative social feedback used in the present study, is reported to be more important to females than to males (Benenson et al., 2013). Benenson et al. showed that females experience more arousal when confronted with exclusion and are faster than males in detecting social exclusion information. Therefore, it could be argued that the negative social feedback in this task could have received more attention from females, who perceived it as being more relevant and having more impact. In this way, for women the amount of attention paid to negative social feedback stimuli might have become more equal to the amount of attention paid to the more rewarding expected positive feedback stimuli. This might have led to smaller or even absent differences between the different conditions in the task. Our results with respect to gender differences confirmed this by showing that females, when tested separately, showed no condition effects.

Cardiac deceleration was largest for unexpected “do not like” judgments, which was completely in line with the previous studies that had measured cardiac measures in this task (Dekkers et al., 2015; Moor, Crone, & van der Molen, 2010; van der Veen et al., 2014). This stronger deceleration has been related to activation of the dACC (Moor, Crone, & van der Molen, 2010) and the role of this structure as a neural alarm system implicated in processing cues of social pain (Eisenberger & Lieberman, 2004). The cardiac response seems to have been very consistent over different experiments and is apparently less sensitive to gender differences than are P3 amplitudes. However, we did find some subtle effects of gender on the cardiac response, suggesting somewhat stronger task effects in males.

With regard to our oscillatory measures, we found that both measures were useful and could differentiate between conditions. As predicted, theta power was strongest for incongruent, unexpected social feedback stimuli. Since theta power is strongly related to the FRN (Cohen et al., 2007), this result is in line with previous studies reporting a larger FRN for incongruent judgments (Dekkers et al., 2015; Van der Molen et al., 2013). Theta oscillations were not dependent on gender, which can be seen as additional evidence that P3 amplitudes and theta oscillations reflect different aspects of the task.

As predicted, delta power was largest for expected “like” judgments. Our hypothesis was based on earlier studies in which it had been found that delta power is higher for rewards than for punishments (Cohen et al., 2009) and our interpretation of the expected “like” judgments as the most rewarding stimuli in this paradigm (van der Veen et al., 2014). Delta power was strongly associated with P3 amplitude, which is in line with earlier studies relating P3 amplitude to delta oscillations (Basar et al., 1984).

Depressive symptoms as measured with the BDI did not influence prediction bias or psychophysiological responses in this experiment. This was somewhat unexpected, especially for the prediction bias. According to the cognitive theory of Beck (1979), depressed patients have a negative cognitive bias. In the social judgment task, this would have resulted in a more negative prediction bias for participants scoring higher on the BDI. The lack of a correlation is possibly related to the subclinical sample used in the present study, with relatively low BDI scores. Another possible explanation is that we examined the association between depressive symptoms and social rejection in only one direction. In our study, we examined the level of preexisting depressive symptoms and related this to the physiological responses, but we did not examine the effect of social rejection on the development of depressive symptoms. According to the model of Slavich et al. (2010), social rejection can be seen as a stressful event that can lead to the development of depressive symptoms. Interpreted in this way, more depressive symptoms do not necessarily predict a stronger response to negative social feedback stimuli, but more social rejection does predict more depressive symptoms.

Neuroticism was only weakly related to prediction bias and the cardiac response to expectations to be liked. The finding that more negative expectations go together with higher neuroticism scores was in line with our hypotheses. As we stated before, neuroticism can be seen as a vulnerability factor for major depressive disorder (MDD), and MDD is thought to be associated with a negative bias (Beck, 1979). The association between a smaller cardiac deceleration and higher neuroticism scores was unexpected. We hypothesized that participants scoring high on neuroticism questionnaires would be more sensitive to unexpected rejection, and therefore would show stronger decelerative cardiac responses to this type of stimulus. A possible explanation for this finding is that a social judgment can be seen as a stressful event, especially when a positive judgment is predicted. As we argued in our previous study (van der Veen et al., 2014), people might only get really involved in the task when they predict that a person likes them, and only in this case would the task become stressful. Previous research has shown that people who score higher on neuroticism show a blunted cardiovascular stress response (Bibbey, Carroll, Roseboom, Phillips, & de Rooij, 2013; Chida & Hamer, 2008). The present finding of a smaller cardiac deceleration for people scoring higher on neuroticism in our task can possibly be seen as such a blunted cardiovascular response to stressful events. An anonymous reviewer suggested that the blunted cardiovascular response might also be related to the association between neuroticism and prediction bias. Participants that score high on neuroticism show a lower bias, and this might possibly influence the cardiac response to the less frequent predictions of positive judgments. However, it should be noted that less frequent events most often lead to stronger cardiac decelerations (e.g., Guerra, Sanchez-Adam, Miccoli, Polich, & Vila, 2016), and therefore we think the lower probability of events cannot be used as a straightforward explanation of the found association.

Besides the obvious strengths of a relatively large sample size and multidimensional outcome measures, this study has some limitations. The first limitation is that we only tested subclinical participants, which could be seen as one of the most important possible causes for a lack of strong correlations between the subjective, performance, and psychophysiological measures. A second limitation was that both depressive symptoms and neuroticism were measured with self-report inventories, which could have led to socially desirable answers, and therefore to noisy estimations of depressive symptoms and neuroticism scores. A final limitation is that the statistical results were not corrected for multiple comparisons, and therefore the weak correlations between our clinical variables and physiological measures should be evaluated with caution.

To summarize, we showed that the various measures used in this experiment were sensitive to different aspects of the task. P3 amplitude was most sensitive to the rewarding properties of the predicted “like” judgment, whereas cardiac deceleration was more sensitive to the “heart-brake of social rejection” (Moor, Crone, & van der Molen, 2010), as reflected in the large decelerative response to unexpected “do not like” judgments. Theta oscillatory power, on the other hand, was most sensitive to the congruence of prediction and judgment. This study also showed that the large individual differences in predication bias and psychophysiological responses in the social judgment task are only weakly related to neuroticism and not related to depressive symptoms. Future research should focus on different factors in order to explain the large individual differences found in this task. One important individual difference in the response to social feedback that should be explored further in future studies is gender, since various physiological responses to social feedback were modulated by gender in this study.
